# Aberrant Expression of *EZH2 *in Pediatric Patients with Myelodysplastic Syndrome: A Potential Biomarker of Leukemic Evolution

**DOI:** 10.1155/2019/3176565

**Published:** 2019-12-10

**Authors:** Teresa de Souza Fernandez, Tatiana Fonseca Alvarenga, Elaiza Almeida Antônio de Kós, Viviane Lamim Lovatel, Rita de Cássia Tavares, Elaine Sobral da Costa, Cecília de Souza Fernandez, Eliana Abdelhay

**Affiliations:** ^1^Bone Marrow Transplantation Center, National Cancer Institute (INCA), 20230-130 Rio de Janeiro, RJ, Brazil; ^2^Pathological Anatomy Department (DIPAT), National Cancer Institute (INCA), 20230-130 Rio de Janeiro, RJ, Brazil; ^3^Pediatrics Department, Faculty of Medicine, Federal University of Rio de Janeiro, 21941-590 RJ, Brazil; ^4^Mathematical and Statistical Institute of Federal Fluminense University (UFF), 24020-140 Niterói, RJ, Brazil

## Abstract

Pediatric myelodysplastic syndrome (MDS) is an uncommon disease and little is known about the molecular alterations of its development and evolution to acute myeloid leukemia (AML). The *Enhancer of Zeste Homolog 2* (*EZH2*) is the catalytic subunit of Polycomb repressive complex 2 (PCR2). It is a histone methyltransferase, that targets lysine 27 of histone 3. This methylated H3–K27 is usually associated with the silencing of genes that are involved in fundamental cellular processes, such as cell proliferation and differentiation. There are only few studies showing the status of *EZH2* expression in patients with MDS and they were performed in adult MDS patients. The aim of this study was to analyze the *EZH2* expression in pediatric patients with MDS and its association with karyotypes and evolution to acute myeloid leukemia (AML). We conducted the first study of *EZH2* expression in pediatric patients with MDS. Considering the *EZH2 *expression levels in 42 patients and 17 healthy pediatric donors, it was possible to define three groups of expression in patients: low, intermediate, and high. The intermediate level encompassed patients with normal karyotypes, low level included patients with monosomy 7 and del(7q) and high level included patients with trisomy 8 and del(11q) (*p* < 0.0001). Comparing the leukemic evolution, the low expression group presented disease evolution in 100% (8/8) of the cases, the intermediate expression group showed disease evolution in 4.34% (1/23) and in the high expression group, 63.63% (7/11) patients showed evolution from MDS to AML (*p* < 0.0001). It is important to note that low and high *EZH2* expression are associated with leukemic evolution, however low expression showed a stronger association with evolution from MDS to AML than the high expression. Our results suggest a scale of measure for *EZH2 *expression in pediatric MDS, where aberrant *EZH2 *expression may be a potential biomarker of disease evolution.

## 1. Introduction

Myelodysplastic syndrome (MDS) is a heterogeneous group of clonal hematological neoplasms with a variable clinical features and diverse genetic and epigenetic alterations. The major clinical MDS characteristics are ineffective hematopoiesis, dysplasias, peripheral cytopenias, and an increased risk of transformation to acute myeloid leukemia (AML) [1]. MDS is viewed as a disease of adults, particularly the elderly patients. Pediatric MDS is a rare disease, accounting for less than 5% of childhood hematologic malignancies [2–4].

In pediatric patients with MDS, the clonal cytogenetic alterations can be detected in 50–70% of the cases [5]. The most frequent chromosomal abnormalities are monosomy 7 and del(7q) [3]. The cytogenetic evaluation of a bone marrow sample from patients with MDS has become an integral part of clinical care [5–7]. However, there is a percentage of patients with normal karyotypes. So, it is important a molecular characterization of genetic and epigenetic alterations associated with the evolution of the disease, which could help predict prognosis.

Most knowledge about molecular alterations in MDS was acquired from studies in adult patients and it has been observed the importance of epigenetic alterations in the MDS pathogenesis, since it is the most responsive disease to DNA methylation inhibitors drugs [8]. Epigenetic modifications play important biological roles because they regulate gene expression. There are two main epigenetic modifications: the DNA methylation and the chromatin modification, which are frequently associated to transcriptional gene repression. Among chromatin modifiers, Polycomb Group (PcG) proteins have been established as classical players of epigenetic regulation [9]. PcG proteins contain two core complexes: the maintenance polycomb repressive complex 1 (PCR1) and the initiation polycomb repressive complex 2 (PCR2). The *Enhancer of Zeste Homolog 2* (*EZH2*) is a histone methyltransferase. It is the catalytic subunit of PCR2 for tri-methylation of histone 3 at lysine 27 (H3K27me) by SET domain in its C-terminus, which silences target genes involved in various biological functions as cell cycle, cell proliferation and differentiation. PcG proteins are important epigenetic regulators and critical factors of pluripotency and differentiation of stem cells as well as aberrant gene expression during the malignant transformation [10].

Overexpression of *EZH2* is frequently observed in many cancer types, including prostate, breast, bladder, ovarian, lung, liver, gastric esophageal, pancreatic cancer, melanoma, and osteossarcoma. This overexpression in solid tumors is correlated with higher proliferation and poor prognosis. So, there are results suggesting that *EZH2* has a critical role in cancer progression and an epigenetic therapy that pharmacologically targets *EZH2* may constitute a novel approach to the treatment for some types of cancer [11, 12].

The biological functions of *EZH2* in different tumor cells are under intense investigation. In MDS and AML, both overexpression and loss-of-function mutations of *EZH2* gene suggest that it can function as an oncogene or as a tumor suppressor gene, respectively [10]. There are only few studies showing the status of *EZH2* expression in patients with MDS. These studies were done in adult patients. Xu and colleagues studied for the first time the Polycomb expression genes including *EZH2*. In this study, it was observed that overexpression of the *EZH2* gene is common in MDS and indicates poor prognosis [13]. However, Cabrera and colleagues observed underexpression of *EZH2 *and its association with chromosome 7 alterations [−7 and del(7q)] and poor prognosis in MDS [14].

The functions of *EZH2* and its mapping to the critical region for malignant myeloid disorders suggest that the *EZH2* gene may be involved in the pathogenesis of 7q35-q36 alterations [15]. Until now, there are no studies showing the expression of *EZH2* in pediatric patients with MDS. Therefore, the aim of this study was to analyze the *EZH2* gene expression in pediatric patients with MDS, concerning their association with karyotypes, MDS subtypes and evolution from MDS to AML, giving new insights into pediatric MDS pathogenesis.

## 2. Materials and Methods

### 2.1. Patients and Controls

Bone marrow cells were obtained from 42 patients with pediatric MDS between 2007 and 2016. The patients included 27 boys and 15 girls, with ages between 5 months and 18 years (the mean age was 8 years). The patients were diagnosed at the Hematology/Oncology Units of hospitals in Rio de Janeiro, Brazil: National Cancer Institute (INCA-RJ) and Martagão Gesteira Institute of Pediatrics and Child Development (IPPMG-UFRJ). Diagnosis and classification were done according to the revised criteria of Hasle and colleagues, 2003 and 2016 [16, 17]. Twenty-two patients were classified as refractory childhood cytopenia (RCC), fifteen as refractory anemia with excess of blasts (RAEB), and five as refractory anemia with excess of blasts in transformation (RAEB-t). None of the patients had been previously treated for a malignancy. Healthy bone marrow samples were obtained from 17 pediatric donors of bone marrow transplant (the mean age was 12 years). This study was approved by the Ethics Committee of the National Cancer Institute (INCA, Rio de Janeiro, Brazil) and all procedures performed followed the bioethics standard, according to resolution 466/12 of Health National Committee.

### 2.2. Cytogenetic Analysis

Karyotypes of bone marrow cells from the 42 pediatric patients with MDS were obtained from cultures in RPMI 1640, with 20% fetal calf serum (GIBCO) at 37°C for 24 hours. Cell cultures were pulsed with colcemid to a final concentration of 0.05 *µ*g/mL at the final hour of incubation. Cells were subsequently harvested by standard procedures (hypotonic shock: 0,075 M) and fixed in methanol: acetic acid (3 : 1). GTG-banding was performed. Chromosomes were identified and arranged according to the International System for Cytogenetic Nomenclature, 2016 [18]. Fluorescence “in situ” hybridization (FISH) analyses were performed using dual color probe for chromosome 11 (LSI MLL dual color break apart rearrangement probe) according to the manufacturer's protocol, to confirm the del(11)(q23) with the allelic loss of *MLL/KMT2A*. We used the samples of cytogenetic cultures.

### 2.3. Analysis of EZH2 Gene Expression by Real-Time Quantitative PCR

Analyses of *EZH2* mRNA level alterations in 42 pediatric patients with primary MDS and 17 healthy pediatric controls were carried out by real-time quantitative polymerase chain reaction (qRT–PCR). Total mRNA was obtained from the 42 pediatric patients with MDS and 17 healthy individuals (donors) by TRIzol reagent (*Life Technologies*), according to the manufacturer's protocol and stored at −70°C. Two micrograms of total RNA was submitted to genomic DNA digestion with DNAse amplification grade I (*Life Technologies*) to remove genomic DNA contaminant. The RNA was transcribed reversely to complementary DNA (cDNA) with Superscript II Reverse Transcriptase (*Life Technologies*) and Oligo-dT18 (*Life Technologies*) kits. Reactions were performed in 10 *µ*l with final concentration of 1 × of Rotor-Gene SYBR Green PCR Kit (Qiagen); 0.5 *µ*M of each forward/reverse primers and completed 2.5 *µ*l of cDNA diluted five folds. Reactions were carried out in Rotor Gene Q thermocycler (Qiagen), with hot-start stage step of 10 minutes at 95°C followed by 45 cycles of 20 seconds at 95°C, 30 seconds at 60°C, and 30 seconds at 72°C. The dissociation curve was used to determine the PCR efficiency, specificity of amplification and primer dimer formation. *Β-actin* mRNA levels were used as a reference for normalization. The following primers sequence used were: *EZH2* Forward 5′-TTGTGACAGTTCGTGCCCTTGT-3′ and *EZH2* Reverse 5′-TGCTTGGTGTTGCACTGTTGCTT-3′ *Β-actin* Forward 5′-TTCCTTCCTGGGCAT GGAGTC-3′ and *Β-actin* Reverse 5′-AGACAGCACTGTGTT GGCGTA-3′. The relative expression levels of *EZH2* gene were calculated using the ∆∆C_T_ method.

### 2.4. Statistical Analysis

The statistical difference between *EZH2 *expression in pediatric patients and donors (pediatric healthy individuals) was analyzed by Mann–Whitney test. This test was also used in the analyses of age groups (children versus adolescents), MDS subtypes (initial stage, RCC, versus advanced stages, RAEB and RAEB-t), karyotypes (normal karyotypes versus abnormal karyotypes) and *EZH2 *expression in donors and low *EZH2* expression patients, donors and intermediate *EZH2* expression patients and donors and high *EZH2* expression patients. The Kruskal–Wallis test was used to verify the statistical difference between three groups defined according to the level of *EZH2* expression (low, intermediate and high). We also analyzed the levels of *EZH2* expression and the evolution of disease (from MDS to AML) through the chi-square test with Yates correction. The difference between low expression group and high expression group was analyzed by Fisher's exact test. A value of *p* < 0.05 was considered significant in all analyses.

## 3. Results

### 3.1. Clonal Chromosomal Abnormalities in Pediatric Patients with Primary MDS

**Figure 1:**
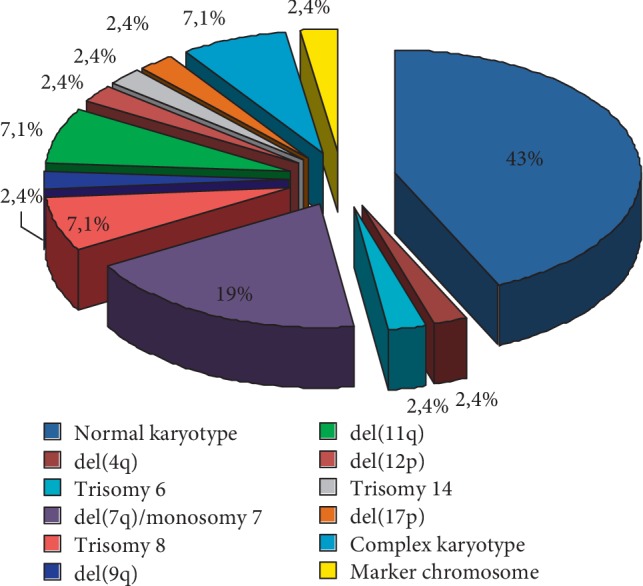
Frequency of chromosomal abnormalities in pediatric patients with primary MDS.

Among a total of 42 pediatric patients with primary MDS, clonal chromosomal abnormalities were detected in 24 patients (57%). The frequency of the chromosomal abnormalities in pediatric patients with primary MDS is shown in (Figure 1). The distribution of abnormal karyotypes according to MDS subtypes was: 23% (5/22) in RCC, 93% (14/15) in RAEB, and 100% (5/5) in RAEB-t. Cytogenetic results showed that patients with RCC presented normal karyotypes or single abnormalities as: del(4q), del(9p), del(11q), del(12p), and +mar. In the RAEB and RAEB-t subtypes, we observed single chromosomal abnormalities as +6, del(7q), −7, +8, del(11q), del(17p), +14, and complex karyotype. In the cases of del(11q), FISH analyses were performed showing the del(11)(q23) with allelic loss of *MLL/KMT2A*. The clinical and cytogenetic characteristics in pediatric patients with primary MDS is shown in Table 1.

### 3.2. Analysis of EZH-2 Gene Expression in Pediatric Patients with Primary MDS

**Figure 2:**
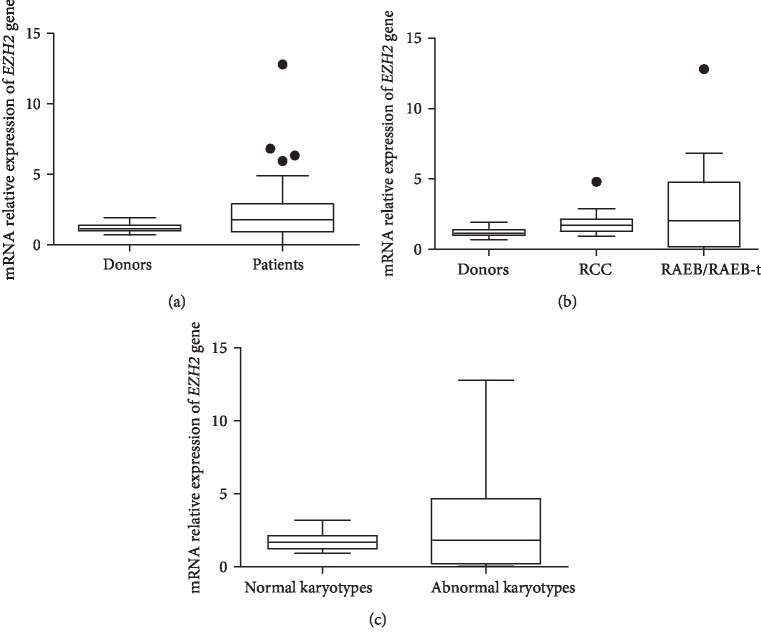
*EZH2* expression by qRT-PCR in pediatric patients with MDS. (a) *EZH2* expression from samples of healthy pediatric donors and pediatric patients with MDS. (b) Healthy pediatric donors and MDS subtypes. (c) Normal karyotypes versus abnormal karyotypes.

The analysis of *EZH-2* gene relative expression levels in 42 pediatric patients with primary MDS showed a higher expression when compared to the controls (donors), which led to a statistically significant analysis (*p* < 0.04) (Mann–Whitney test) (Figure 2(a)). Analyzing the *EZH-2* relative expression levels according to the subtypes of MDS, being 22 patients classified at initial stage (RCC) and 20 patients classified at advanced stages (RAEB and RAEB-t), we found no statistical significance (Figure 2(b)). The association of *EZH2 *relative expression levels between patients with normal karyotypes and abnormal karyotypes did not show a statistical significance (Mann–Whitney test) (Figure 2(c)).

**Figure 3:**
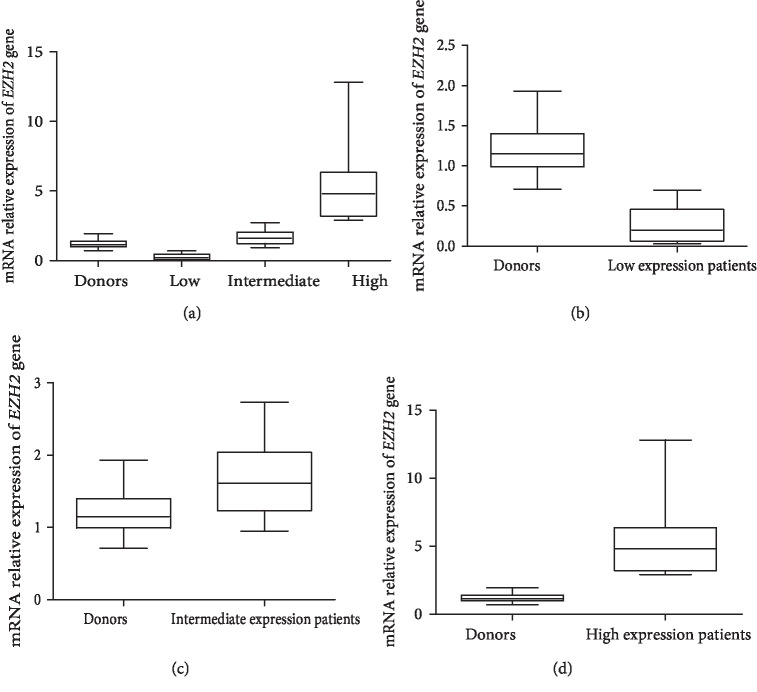
*EZH2* expression by qRT-PCR in pediatric patients with MDS. (a) *EZH2* expression levels: low, intermediate, high and donors. (b) Donors vs low expression patients. (c) Donors vs intermediate expression patients. (d) Donors vs high expression patients.

However, it is very interesting to note that analyzing the distribution of the expression levels of *EZH2* in patients and donors, it is clearly observed that there is a heterogeneous distribution in patients, while the distribution of expression levels in controls is more homogeneous. From this result, it was possible to define three distinct groups of *EZH2* expression. This analysis was based mathematically on quartiles [19]. Since the distribution of the *EZH2* expression in patients is skewed, we used the median as central measure and quartiles as dispersion measures, according to Zar, 2010 [19]. We found 1.8 for the median. The first quartile is 1 and the third quartile is 2.85. We divided the *EZH2 *expression in patients into three groups: low, intermediate, and high. In order to compare *EZH2* expression of patients and donors, we also used the median and quartiles for the donors. In normal individuals (donors), the median was 1.15 and according to the classes of *EZH2* expression, the median at low level was 0.2, for intermediate level the median was 1.61 and for high expression, the median was 4.81 (Figure 3(a)). We observed a significant difference between the *EZH2* expression in donors and in low (*p* < 0.0001) (Figure 3(b)), intermediate (*p* < 0.0057) (Figure 3(c)) and high (*p* < 0.0001) (Figure 3(d)) *EZH2* expression groups. It was possible to suggest a scale of *EZH2* expression, where patients with *EZH2* expression belonging to the real interval [0,1) were classified in the low group, the real interval [1,3) represented the intermediate group and the real interval [3,13) represented the high group. We have *n*=8, *n*=23, and *n*=11 as the number of patients in each group, respectively (Table 2).

**Figure 4:**
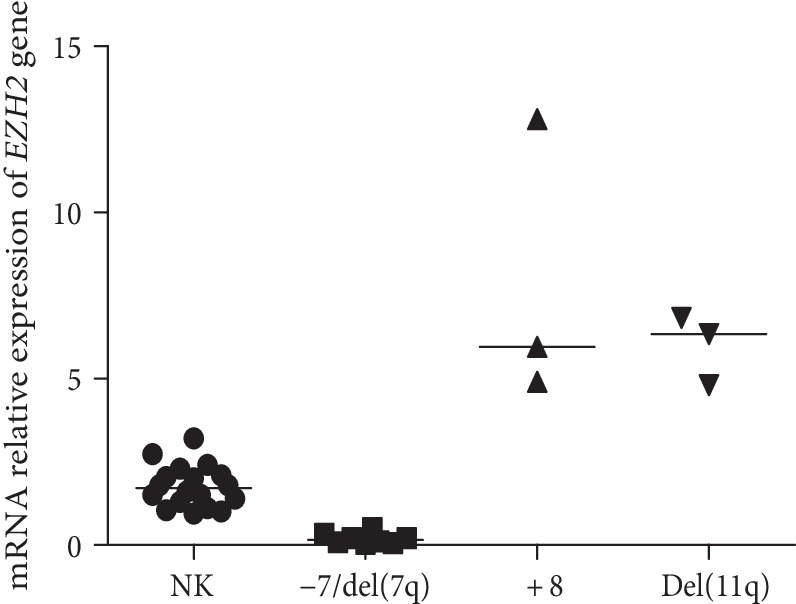
*EZH2* expression levels with specific karyotypic patterns: normal karyotypes (NK), −7 and del(7q), +8, del(11q).

Then, we analyzed the association of *EZH2* expression levels with specific karyotypic patterns. Our results showed that intermediate *EZH2* expression level encompassed patients with normal karyotypes (*n* = 18), low level of expression included patients with monosomy 7 and del(7q) (*n* = 8) and high level of expression included patients with trisomy 8 (*n* = 3) and del(11q) (*n* = 3). We observed a statistical difference between these groups (*p* < 0.0001, Kruskal–Wallis test) (Figure 4). It is important to notice that karyotypes can be categorized using the scale of *EZH2* expression suggested in this work. More precisely, none patients with monosomy 7 and del(7q) was in high expression group and none of patients with trisomy 8 and del(11q) was in low expression group (Table 2). It is also important to observe that we found other karyotypes, as we can see in Figure 1, but we considered normal karyotypes, monosomy7 and del(7q) and trisomy 8 and del(11q) for the association analysis with *EZH2* expression, because of the number of patients in these cytogenetic groups (Table 2).

We also investigated the *EZH2* relative expression levels and the evolution of the disease according to the three *EZH2* expression groups. The low expression group presented evolution of the disease in 100% of cases (8/8 patients), the intermediate expression group showed disease evolution in 4.34% patients (1/23) and in the high expression group, 63.63% patients (7/11) showed evolution from MDS to AML. We verified through the chi-square test with Yates correction a statistical significance in this result (*p* < 0.001). It is important to note that the difference between low expression group (8/8) and high expression group (7/11) is not significant statistically (*p*=0.085, Fisher's exact test). This result is very important, because it demonstrates that both groups, the low *EZH2 *expression group and the high *EZH2 *expression group, are associated with evolution from MDS to AML. Moreover, our result suggests that it seems that the low expression group presents a more elevated risk of progression to AML, since there was a 100% of disease evolution in the cases studied. Our results suggest that this scale of measure for *EZH2* expression in pediatric MDS can give us a better understanding of the evolution from MDS to AML (Table 2). The associations of *EZH2* gene expression with clinical features, karyotypes and leukemic evolution in pediatric patients with primary MDS and *p* values are shown in Table 3.

## 4. Discussion

The molecular pathogenesis of pediatric MDS remains poorly understood due to its rarity, high heterogeneity and complexity of the disease. Until now, few studies have evaluated the expression of *EZH2* in MDS. Xu and colleagues evaluated the expression of the *EZH2 *gene in bone marrow samples from 54 adult patients with MDS using the qRT–PCR methodology. The authors verified increased expression of *EZH2* and two other Polycomb genes, *BMI1* and *RING1*, in these patients. In this study, it is interesting to note that cytogenetic analysis showed, as main numerical chromosomal abnormality, the trisomy 8 and it was not detected the alterations in chromosome 7 (monosomy 7/del 7q). The authors suggested that increased expression of Polycomb genes, including the *EZH2*, is an event related to poor prognosis in MDS [13]. On the other hand, Cabrero and colleagues studied the expression of *EZH2* in 78 adult patients with MDS. According to this study, patients who presented alterations in chromosome 7, such as monosomy 7 and 7q deletion, showed a significantly lower level of expression when compared to controls, diploid patients and other chromosomal abnormalities [including del (5q), +9, +16, +8, del(11q), −Y, t(7;21), inv3, del (20q)]. In this study, the underexpression of *EZH2* and alterations of chromosome 7 were associated with poor prognosis [14]. McGraw and colleagues studied EZH2 protein expression by immunohistochemistry in 33 MDS patients. It was observed that EZH2 expression was significant lower in −7 and del(7q) compared to those patients without these chromosomal alterations [20]. Xu and colleagues showed that the genomic loss of *EZH2* [−7 and del(7q)] leads to low *EZH2* expression in MDS and it is associated with shorter survival and increased AML transformation [21]. However, Sashida and colleagues showed that EZH2 loss promotes development of MDS, but attenuates its predisposition to leukemic transformation [22]. Thus, it is possible to note that studies regarding the role of *EZH2* in MDS in adult patients are still controversial.

We conducted the first study of *EZH2* expression in pediatric patients with MDS. In our study, we observed a statistically significant difference in the expression of *EZH2* between the group of patients and the group of controls. Patients showed an *EZH2* expression distribution ranging from 0.03 to 12.8 and controls showed an *EZH2* expression distribution ranging from 0.71 to 1.93. So, the *EZH2* expression distribution observed in patients is more heterogeneous than that in the controls. From this observation, it was possible to define three distinct groups of expression (low, intermediate, and high) using the median as central measure for the study. These results suggest a scale of measure for *EZH2* expression in pediatric MDS.

Analyzing the expression levels of *EZH2* in pediatric patients with MDS and the evolution of disease, we observed that the low expression group presented evolution of the disease in 100% of the cases, the intermediate expression group showed disease evolution in 4.34% patients, and in the high expression group, 63.63% patients showed evolution from MDS to AML. This result was significant (*p* < 0.0001). Thus, our results suggest that aberrant expression of *EZH2* is associated with leukemic transformation, being a biomarker of disease evolution. It is important to note that low and high expression levels were associated with leukemic evolution; however, low expression showed a stronger association with evolution from MDS to AML than the high expression.

Regarding the evolution from MDS to AML, Mc Graw and colleagues suggested the EZH2 protein analysis by immunohistochemistry may be a molecular tool for discriminating disease outcome or transformation risk [20]. In our study, we also observed the importance of *EZH2* expression results in MDS. So, studies based on *EZH2* expression may have relevant clinical implications and may be incorporated as an additional laboratory test using also the real-time quantitative polymerase chain reaction (qRT–PCR).

Accumulating studies have demonstrated that *EZH2* participates in various biological processes and displays different modes of action [10]. These results showed that the clinical impact of aberrant *EZH2* expression on the progression of MDS is complex and appears to be associated with other events such as the presence of a specific chromosomal abnormality. This hypothesis may be re-inforced because in MDS there are different molecular evolution pathways associated with specific cytogenetic abnormalities [23, 24]. Based on these results, it is necessary new studies to clarify the signaling pathways in the three groups of *EZH2 *expression and studies involving a larger number of patients. Some studies have been reported the molecular pathways associated with EZH2 [10, 20, 25]. Xu and colleagues demonstrated that genomic loss of *EZH2* contributes to overexpression of the *HOX* gene clusters in MDS by reducing H3K27me3 [21].

Overexpression of *EZH2* has been found in different cancer types [26]. *EZH2* was found to be overexpressed in MDS tumor cells associated with methylation of tumor suppressor gene cyclin dependent kinase inhibitor 2B (*p15^INK4B^*) compared with tumor cells that *p15^INK4B^* is not methylated. This association between *EZH2 *and* p15^INK4B^* was not observed in other PcG genes (*EED*, *SUZ12*, *BMI-1* and *RING1*), suggesting that *EZH2* is involved in the methylation of *p15^INK4B^* gene [13]. Methylation in *p15^INK4B^* and *p16^INK4A^* were observed in pediatric MDS suggesting that these genes may play an important role during evolution from MDS to AML [2]. Recently, Ye and Li demonstrated in a systematic meta-analysis the important role of *p15^INK4B^*in the development, progression, and poor prognosis of MDS [27].

In prostate cancer, there is a positive feedback mechanism between *Myc* and *EZH2, *where *Myc* overexpression is associated with increased *EZH2* and *EZH2* could also induce *Myc* overexpression. *Myc* is a transcription factor that regulates cell proliferation. It has been considered as an important factor of the increase expression of *EZH2* by repressing microRNAs expression (miR26a and miR26b). The initiation and evolution of prostate cancer is associated with overexpression of *Myc *and* EZH2* [28]. In other tumors, like medulloblatoma, cooperation between *Myc* and *EZH2* was also observed, where higher *Myc* levels were associated with increased *EZH2* and pharmacological blockade of *EZH2* is a potential therapeutic strategy for aggressive medulloblatoma [29]. Different mechanisms are involved in *c-Myc* deregulation expression in hematological neoplasms, such as chromosome rearrangements, amplification and epigenetic mechanisms. Increased expression of *EZH2* by *c-Myc* has been described in AML. As observed in solid tumors, in hematological neoplasms, there is also an association between *c-Myc* deregulation and *EZH2* overexpresion [30]. In chromosome 8, region q24 is located the gene *cMyc. *In our study, we observed an association of trisomy 8 and an overexpression of *EZH2*, associated in evolution of disease. It was already observed that trisomy 8 cells in MDS have amplification intrachromosomally of *c-Myc* [31]. Therefore, it is possible, like in other types of cancer that *c-Myc* and *EZH2 *overexpression cooperate in the pathogenesis of MDS, mainly during evolution to AML.

It has been described that imbalance between Polycomb (PcG) and Trithorax (TrxG) expressions is involved in development of diseases, as cancer. The PcG proteins usually maintain the repression of gene expression and TrxG proteins act in the opposite way [32]. *KMT2A* (*MLL*) gene is located in region q23 in chromosome 11 and it is an important member of TrxG family, involved in pediatric acute leukemia pathogenesis [33]. Abnormalities in the balance between PcG and TRxG may be associated with leukemia pathogenesis [32]. In our study, we found an association of del(11q), involving the loss of *KMT2A* allele, and an overexpression of *EZH2*. Our results suggest that a possible mechanism involved in the evolution of disease in this case may be related with an imbalance between the expression of *EZH2* and *KMT2A* genes.

The *EZH2* aberrant expression in cancer cells may result from different mechanisms involved in poor prognosis with evolution of disease. Therefore, further investigation is necessary to a better understanding of the mechanisms associated with low and high expression of *EZH2* during the pathogenesis of MDS.

*EZH2* is a master regulator of chromatin and accumulated evidence suggests that it is involved in aberrant transcriptome in cancer cells. Thus, EZH2 is a potent target for cancer therapy and EZH2 inhibitors have been under pre-clinical and clinical investigations [25]. However, as demonstrated in other studies, the *EZH2* may have tumor suppressive and oncogenic functions in MDS and in AML. It is also demonstrated that *EZH2* has a dual role in the same disease, acting in different phases of the AML. This dual function has potential therapeutic implications [34, 35]. Analyzing our results of *EZH2* expression in pediatric patients with MDS, it is also possible to suggest the dual role of *EZH2* and this result has clinical implications, regarding the evolution of disease and also to aid the choice of the treatment, reinforcing the importance of incorporate additional laboratory tests to analyze the *EZH2 *expression. Since this study was the first to investigate the association of aberrant expression of *EZH2* with karyotypes and disease evolution in pediatric patients with MDS, a more number of studies are necessary to confirm our results and the relevant contribution of *EZH2* in the pathogenesis of pediatric MDS.

## 5. Conclusions

We conducted the first study of *EZH2* expression in pediatric patients with MDS. Considering the *EZH2* expression levels in patients and healthy pediatric donors, it was possible to define three groups of expression in patients: low, intermediate, and high. The intermediate level encompassed patients with normal karyotypes, low level included patients with monosomy 7 and del(7q) and high level included patients with trisomy 8 and del(11q) (*p* < 0.0001). Comparing the leukemic evolution, it was important to note that low and high *EZH2* expression were associated with leukemic evolution. However, the low expression showed a stronger association with evolution from MDS to AML than the high expression. Our results provides new insights about the pediatric MDS pathogenesis with the focus on the aberrant expression of *EZH2*, suggesting a scale of measure for *EZH2* expression in pediatric MDS, where aberrant *EZH2* expression may be a potential biomarker of disease evolution.

## Figures and Tables

**Table 1:** Clinical and cytogenetics characteristics of pediatric patients with primary MDS.

Clinical characteristics	Number of Patients (%)
*Age*	
Mean of age: 8 years old (range from 5 months to 18 years)	42 (100%)
*Sex*	
Male	27 (64%)
Female	15 (36%)
*MDS subtypes*	
Initial subtype: RCC	22 (52%)
Advanced subtypes	
RAEB	15 (36%)
RAEB-t	5 (12%)
*Karyotypes*	
Normal	18 (43%)
Abnormal	24 (57%)
del(4q)	1 (2.4%)
Trisomy 6	1 (2.4%)
del(7q)	3 (7.1%)
Monosomy 7	5 (12%)
Trisomy 8	3 (7.1%)
del(9q)	1 (2.4%)
del(11q)	3 (7.1%)
del(12p)	1 (2.4%)
del(17p)	1 (2.4%)
Trisomy 14	1 (2.4%)
Marker chromosome	1 (2.4%)
Complex karyotype	3 (7.1%)

**Table 2:** Scale of measure for *EZH2* expression in pediatric MDS.

Scale of expression level of EZH2	[0,1)^∗^	[1,3)^∗∗^	[3,13)^∗∗∗^
Defined groups	Low	Intermediate	High
Karyotypes	Monosomy 7 and del(7q)	Normal karyotypes	Trisomy 8 and del(11q)
Evolution MDS→AML (%)	100%	4.34%	63.63%

Notation in the form of ^∗^[0,1) to indicate the numbers between 0 and 1; ^∗∗^[1,3) to indicate the numbers between 1 and 3; ^∗∗∗^[3,13) to indicate the numbers between 3 and 13.

**Table 3:** Association of *EZH2* gene expression with clinical features, karyotypes and leukemic evolution in pediatric patients with primary MDS.

Parameter	*EZH2 *gene expression (median)		*p* value
*Age of patients*			
< 12 years old (children), *n*=32	1.8		*p* < 0.4
> 12 years old (adolescents), *n*=10	2.7		
*Pediatric patients* (*n*=42)	1.8		*p* < 0.04
*Pediatric healthy individuals* (*n*=17)	1.15		
*MDS subtypes*			
Initial stage (RCC) (*n*=22)	1.7		*p* < 0.9
Advanced stages (RAEB/RAEB-t) (*n*=20)	2.9		
*Karyotypes*			
Normal (*n*=18)	1.7		*p* < 0.8
Abnormal (*n*=24)	1.8		
*Specific chromosomal pattern*			
Normal karyotypes (*n*=18)	1.7		*p* < 0.0001
−7 and del(7q) (*n*=8)	0.2		
+8 (*n*=3)	5.96		
del(11q) (*n*=3)	6.35		
*Levels of EZH2 expression*			
Low (*n*=8)	0.2		*p* < 0.0001
Intermediate (*n*=23)	1.61		
High (*n*=11)	4.81		

*Donors versus levels of EZH2 expression (groups)*	*Donors*	*Groups*	
Donors (*n*=17) vs. Low expression patients (*n*=8)	1.15	0.2	*p* < 0.0001
Donors (*n*=17) vs. Intermediate expression patients (*n*=23)	1.15	1.61	*p* < 0.0057
Donors (*n*=17) vs. High expression patients (*n*=11)	1.15	4.81	*p* < 0.0001
	Low (8/8)		
*Evolution of disease (MDS →AML) versus levels of EZH2 expression*	Intermediate (1/23)		*p* < 0.0001
	High (7/11)		
*Evolution of disease (MDS →AML) versus levels of EZH2 expression*	Low (8/8)		*p*=0.085
	High (7/11)		

## Data Availability

The data used to support the findings of this study are included within the article and the original data used to support the findings of this study are available from the corresponding author upon request.
